# The Great Imitator Revisited—A Case of Meningovascular Neurosyphilis Presenting as Acute Ischemic Stroke With Ataxia

**DOI:** 10.1155/crdi/7663789

**Published:** 2026-11-14

**Authors:** Nabin Adhikari, Punita Singh, Mukti Nath Sankhi, Rohit Vashisht, Raj Kumar Thapa, Laxmi Narayan Kamat, Rajiv Sitaula

**Affiliations:** ^1^ Department of Internal Medicine Nepalese Army Institute of Health Sciences College of Medicine Kathmandu Bagmati Nepal; ^2^ Department of Internal Medicine Army Hospital Research and Referral New Delhi Delhi India

**Keywords:** ceftriaxone, cerebellar ataxia, meningovascular syphilis, neurosyphilis, stroke, *Treponema pallidum*

## Abstract

Neurosyphilis is caused by invasion of the central nervous system by *Treponema pallidum*. Although previously considered uncommon in the postpenicillin era, its incidence has increased in recent years, particularly among men who have sex with men, in association with changing sexual practices, improved clinical recognition, and the widespread availability of effective HIV treatment and pre‐exposure prophylaxis (PrEP). Syphilis has historically been described as the “great imitator” because of its varied clinical picture. We report a case of a 57‐year‐old male presenting with acute‐onset cerebellar ataxia and dysarthria initially diagnosed as acute cerebellar stroke. Further evaluation revealed a history of presumed untreated primary syphilis, positive serum treponemal serology, and reactive cerebrospinal fluid venereal disease research laboratory (CSF‐VDRL) test, leading to the diagnosis of meningovascular neurosyphilis. The patient was successfully treated with intravenous ceftriaxone for 14 days with significant clinical improvement. This case highlights the importance of considering neurosyphilis in patients presenting with atypical stroke syndrome or unexplained cerebellar manifestations.

**Summary**
• Key clinical message:
◦ Meningovascular syphilis should be considered in patients who present as atypical stroke or strokes at unusual vascular territory especially where history is suggestive. Strong clinical suspicion, meticulous history taking, and thorough clinical examination can lead toward the correct diagnosis.

## 1. Introduction

Neurosyphilis is an infection of the central nervous system caused by *Treponema pallidum*, occurring in both early and late stages of syphilis. Central nervous system invasion may occur within days to weeks after primary infection, although clinical manifestations may remain latent for years. Traditionally, neurosyphilis is classified into five subtypes: early forms (asymptomatic, meningeal, and meningovascular neurosyphilis) and late forms (general paresis and tabes dorsalis, which typically manifest years or even decades after the initial infection) [1, 2]. Meningovascular syphilis is a form of meningitis involving vasculitis of small and medium‐size arteries in the central nervous system which may causes strokes. Meningovascular syphilis is usually interposed temporally between early and later forms of neurosyphilis typically occurring 1 to 10 years after the primary infection [3]. In recent years, there has been increasing recognition of atypical and oligosymptomatic presentations of neurosyphilis. These forms deviate from classical patterns and often manifest as isolated neurological syndromes including seizures, chronic headaches, cranial nerve involvement, isolated optic atrophy, and cerebellar ataxia [4]. Cerebellar involvement in neurosyphilis is uncommon and can pose significant diagnostic challenges. We present a case of meningovascular neurosyphilis presenting predominantly as acute cerebellar ataxia and dysarthria in an immunocompetent individual without significant comorbidities.

## 2. Case Report

A 57‐year‐old male with no known prior comorbidities presented with imbalance while walking and slurring of speech for three days. The symptoms had a sudden onset and followed a fluctuating course. There was no history of fever, headache, seizures, altered sensorium, limb weakness, or symptoms suggestive of sensory deficits. The patient denied recent alcohol intake.

He was initially evaluated at a health facility where he was diagnosed with acute cerebellar stroke and managed with antiplatelet agents and statins. However, because of persistent symptoms without improvement, he presented to our tertiary care center for further evaluation.

On detailed history, the patient reported an episode of painless genital ulceration two years earlier following unprotected sexual intercourse with a new sexual partner who had multiple sexual partners. The lesion healed spontaneously without medical treatment.

On admission, the patient was conscious and oriented with a Glasgow Coma Scale score of E4V5M6. Vital signs were stable. General physical examination revealed a single well‐defined skin‐colored to pale plaque over the inner aspect of the prepuce without overlying skin changes or inguinal lymphadenopathy. Neurological examination demonstrated dysarthria and prominent cerebellar signs, including dysdiadochokinesia, impaired heel–shin coordination, and broad‐based ataxic gait. Tone, muscle power, deep tendon reflexes, and sensory examination were normal. Romberg test was negative. Other systemic examinations were unremarkable.

### 2.1. Neuroimaging

MRI of the brain showed a patchy area of altered signal intensity appearing on T2WI/FLAIR and restriction of diffusion noted in the left cerebellum (Figure 1).

**FIGURE 1:**
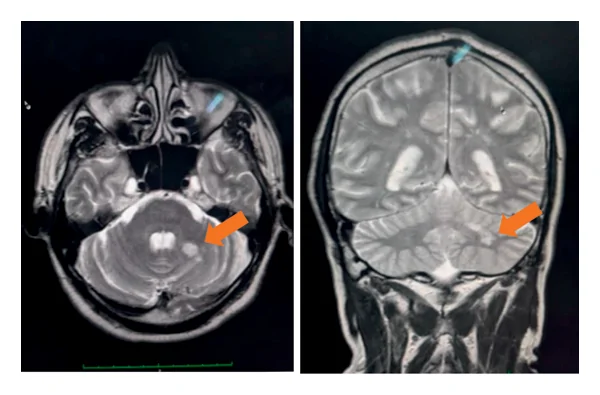
MRI of the brain showing a patchy area of altered signal intensity appearing on T2WI/FLAIR and restriction of diffusion noted in left cerebellum.

Based on the clinical presentation, history of presumed untreated primary syphilis, positive serum TPHA, and reactive CSF VDRL test (Table 1), a diagnosis of neurosyphilis (likely meningovascular with cerebellar involvement) was made. He was managed with injection ceftriaxone for 14 days. He showed marked clinical improvement and discharged home.

**TABLE 1:** Relevant laboratory investigations of the patient.

Baseline investigations	Within normal range
CSF examination	Acellular, protein: 42 mg/dL, glucose: 62 mg/dL (random blood glucose: 130 mg/dL)
CEMRI brain	Suggestive of acute infarct left cerebellum
HIV/HBsAg/anti‐HCV	All nonreactive
Serum VDRL test	Reactive
TPHA test	Reactive
CSF VDRL test	Reactive (1:4)

*Note:* CSF, cerebrospinal fluid; HBsAg, hepatitis B surface antigen; TPHA, treponema pallidum hemagglutination.

Abbreviations: CEMRI, contrast‐enhanced magnetic resonance imaging; HCV, hepatitis C virus; HIV, human immunodeficiency virus; VDRL, Venereal Disease Research Laboratory.

## 3. Discussion

The major clinical categories of symptomatic neurosyphilis include early meningeal and meningovascular and late parenchymatous syphilis. Following initial infection by *Treponema pallidum*, partial immunity develops in the human host which is insufficient to eradicate the organism but leads to latent cardiovascular and neurological infection [5]. Latency from primary infection ranges up to 10–20 years in meningovascular syphilis, 5–20 years for GPI, and 8–30 years for tabes dorsalis [6]. Meningovascular syphilis reflects meningitis together with inflammatory vasculitis of small medium or large vessels. The most common is a stroke syndrome involving the middle cerebral artery [7]. However, unlike the usual thrombotic or embolic stroke syndrome of sudden onset, meningovascular syphilis often becomes manifest after a subacute encephalitic prodrome (headache, vertigo, insomnia, and psychological abnormalities), which is followed by a gradually progressive vascular syndrome, as seen in our case. In 1985, Burke and Schaberg analyzed 30 cases of neurosyphilis diagnosed in the United States between 1970 and 1981. Their findings revealed that 43% of cases presented with classic neurosyphilis symptoms, 14% were asymptomatic, and 43% exhibited nonspecific neurological disorders [5]. Our patient uniquely exhibited a pure cerebellar syndrome with normal CSF proteins and no pleocytosis with CSF VDRL reactive and neuroimaging showing cerebellar infarct likely inflammatory vasculitis. A literature review yields a case report of cerebellar involvement in neurosyphilis presenting with myoclonus, cerebellar ataxia, and executive dysfunction with normal brain imaging but with CSF abnormality [8, 9]. Diagnosis of neurosyphilis is based on the presence of an appropriate clinical syndrome in combination with positive syphilis serology and CSF abnormality (CSF cell count > 5 cells/mm3, elevated protein > 0.45 g/L, and reactive CSF VDRL) [10]. CSF VDRL is a highly specific but insensitive test. If negative, CSF fluorescent treponemal antibody/TPHA should be done due to high sensitivity.

Current treatment guidelines recommend intravenous aqueous crystalline penicillin G 3‐4 million units every 4 hours for 10–14 days as first‐line therapy, and injection ceftriaxone 2 g daily for 10–14 days is considered an effective alternative in situations where penicillin is unavailable or contraindicated [11–13]. In our patient, ceftriaxone was chosen as penicillin was not available. Our patient demonstrated marked clinical improvement following ceftriaxone. This case emphasizes the importance of maintaining a high index of suspicion for neurosyphilis in patients presenting with atypical stroke syndromes, cerebellar ataxia, or unexplained neurological manifestations, particularly when accompanied by a history of high‐risk sexual behavior.

## 4. Conclusion

Neurosyphilis remains an important and reemerging neurological disorder with varied clinical manifestations. Cerebellar ataxia as the predominant presentation is uncommon and can mimic acute cerebrovascular events. Early recognition through detailed history‐taking, targeted serological testing, and cerebrospinal fluid analysis is essential for timely diagnosis and treatment. Neurosyphilis should, therefore, be considered in the differential diagnosis of unexplained cerebellar syndromes and stroke or stroke‐like presentations, even in immunocompetent individuals.

## Funding

No source of funding to declare.

## Ethics Statement

This case report was conducted in accordance with the ethical standards of the Declaration of Helsinki. Institutional ethics committee approval was not required for this single‐patient case report in accordance with institutional policy.

## Consent

Written informed consent was obtained from the patient regarding publication of the data and images.

## Conflicts of Interest

The authors declare no conflicts of interest.

## Data Availability

The data that support the findings of this study are available from the corresponding author upon reasonable request.
